# Predictors of survival in octogenarians after mitral valve surgery for degenerative disease: The Mitral Surgery in Octogenarians study

**DOI:** 10.1016/j.jtcvs.2017.11.027

**Published:** 2018-04

**Authors:** Pierpaolo Chivasso, Vito D. Bruno, Shakil Farid, Pietro Giorgio Malvindi, Amit Modi, Umberto Benedetto, Franco Ciulli, Yasir Abu-Omar, Massimo Caputo, Gianni D. Angelini, Steve Livesey, Hunaid A. Vohra

**Affiliations:** a University Hospitals Bristol, Bristol Heart Institute, Bristol, United Kingdom; b University Hospitals Cambridge, Papworth Hospital, Cambridge, United Kingdom; c University Hospitals Southampton, Wessex Cardiothoracic Centre, Southampton, United Kingdom

**Keywords:** mitral regurgitation, mitral valve, mitral valve surgery, multicenter, octogenarians, AKI, acute kidney injury, BMI, body mass index, CI, confidence interval, COPD, chronic obstructive pulmonary disease, euroSCORE, European System for Cardiac Operative Risk Evaluation, HR, hazard ratio, IABP, intra-aortic balloon pump, LVEF, left ventricular ejection fraction, MiSO, Mitral Valve Surgery in Octogenarians, MR, mitral regurgitation, MVr, mitral valve repair, MVR, mitral valve replacement, OR, odds ratio

## Abstract

**Objectives:**

An increasing number of octogenarians are referred to undergo mitral valve surgery for degenerative disease, and percutaneous approaches are being increasingly used in this subgroup of patients. We sought to determine the survival and its predictors after Mitral Valve Surgery in Octogenarians (MiSO) in a multicenter UK study of high-volume specialized centers.

**Methods:**

Pooled data from 3 centers were collected retrospectively. To identify the predictors of short-term composite outcome of 30 days mortality, acute kidney injury, and cerebrovascular accident, a multivariable logistic regression model was developed. Multiple Cox regression analysis was performed for late mortality. Kaplan–Meier curves were generated for long-term survival in various subsets of patients. Receiver operating characteristic analysis was done to determine the predictive power of the logistic European System for Cardiac Operative Risk Evaluation.

**Results:**

A total of 247 patients were included in the study. The median follow-up was 2.9 years (minimum 0, maximum 14 years). A total of 150 patients (60.7%) underwent mitral valve repair, and 97 patients (39.3%) underwent mitral valve replacement. Apart from redo cardiac surgery (mitral valve repair 6 [4%] vs mitral valve replacement 11 [11.3%], *P* = .04) and preoperative atrial fibrillation (mitral valve repair 79 [52.6%] vs mitral valve replacement 34 [35.1%], *P* < .01), there was no significant difference in terms of any other preoperative characteristics between the 2 groups. Patient operative risk, as estimated by logistic European System for Cardiac Operative Risk Evaluation, was lower in the mitral valve repair group (10.2 ± 11.8 vs 13.7 ± 15.2 in mitral valve replacement; *P* = .07). No difference was found between groups for duration of cardiopulmonary bypass and aortic crossclamp times. The 30-day mortality for the whole cohort was 13.8% (mitral valve repair 4.7% vs mitral valve replacement 18.6%; *P* < .01). No differences were found in terms of postoperative cerebrovascular accident (2% vs 3.1%; *P* = .9), acute kidney injury requiring dialysis (6.7% vs 13.4%; *P* = .12), and superficial or deep sternal wound infection (10% vs 16.5%, *P* = .17; 2% vs 3.1%, *P* = .67, respectively). The final multiple regression model for short-term composite outcome included previous cardiac surgery (odds ratio [OR], 4.47; 95% confidence interval [CI], 1.37-17.46; *P* = .02), intra-aortic balloon pump use (OR, 4.77; 95% CI, 1.67-15.79; *P* < .01), and mitral valve replacement (OR, 7.7; 95% CI, 4.04-14.9; *P* < .01). Overall survival for the entire cohort at 1, 5, and 10 years was 82.4%, 63.7%, and 45.5% (mitral valve repair vs mitral valve replacement: 89.9% vs 70.7% at 1 year, 69.6% vs 54% at 5 years, and 51.8% vs 35.5% at 10 years; *P* = .0005). Cox proportional hazard model results showed mitral valve replacement (hazard ratio, 1.88; 95% CI, 1.22-2.89; *P* < .01) and intra-aortic balloon pump use (hazard ratio, 2.54; 95% CI, 1.26-5.13; *P* < .01) to be independent predictor factors affecting long-term survival. Logistic European System for Cardiac Operative Risk Evaluation did not perform well in predicting early mortality (area under the curve, 0.57%).

****Conclusions**:**

In octogenarians, mitral valve repair for degenerative disease is associated with good survival and remains the gold standard, whereas mitral valve replacement is still associated with significant mortality. Logistic European System for Cardiac Operative Risk Evaluation was unable to predict early mortality in our cohort of patients. Larger international multicenter registries are required to optimize the decision-making process in such a high-risk subgroup.

**Figure:**
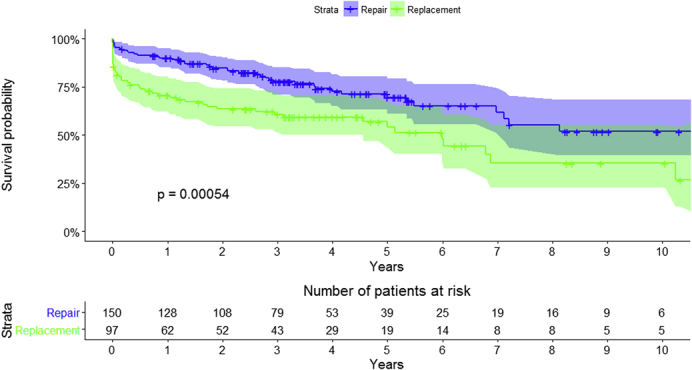
Kaplan–Meier survival curves between the 2 groups (raw data).

**Central Message**

In octogenarians, MVr demonstrated good in-hospital outcomes and provided better survival compared with MVR.

**Perspective**

An increasing number of octogenarians are undergoing valve surgery, and there is a large body of evidence showing that octogenarians derive benefit from cardiac surgery. We demonstrated that MVr is safe and effective even in elderly patients, providing better short-term outcomes and long-term survival compared with MVR.

See Editorial Commentary page 1483.

See Editorial page 1461.

In the past 2 decades, the definition of the “elderly” population in the cardiology literature has evolved: initially age more than 70 years, then more than 75 years, and now more than 80 years.^1^, ^2^ The expectancy and quality of life of the elderly population continue to increase at the cost of a growing prevalence of cardiovascular conditions,^1^, ^2^, ^3^, ^4^ such as mitral valve disease. Thus, an increasing number of octogenarians are referred to undergo cardiac surgical procedures.^4^ Mitral valve repair (MVr) is the treatment of choice for severe mitral regurgitation (MR) in the general population, because it has been shown to provide a significant survival benefit over both medical treatment and mitral valve replacement (MVR).^5^, ^6^, ^7^, ^8^, ^9^, ^10^, ^11^ However, the feasibility and efficacy of mitral repair in very elderly patients are more controversial.^12^ Overall, there is a large body of evidence showing that octogenarians derive benefit from cardiac surgery.^13^, ^14^, ^15^, ^16^, ^17^, ^18^, ^19^, ^20^, ^21^, ^22^, ^23^, ^24^, ^25^, ^26^ In the last decade, several studies have shown that cardiac surgical procedures performed in elderly patients, in otherwise good physical and mental health, can improve their mortality, morbidity, and quality of life.

Transcatheter mitral technologies are emerging as a viable option to treat high-risk and inoperable patients with mitral valve disease.^27^, ^28^, ^29^, ^30^, ^31^, ^32^ These have shown promising early outcomes, especially in terms of procedural safety, and have being increasingly used in otherwise fit octogenarians requiring intervention for degenerative mitral valve disease. To this end, a clear understanding of the risks and benefits of mitral surgery in octogenarians in the current era is needed for rational decision-making regarding the best management option in this cohort of patients.

The purpose of this study was to determine the survival and determinants of its predictors after surgical MVr and MVR in octogenarians in a multicenter UK study, Mitral Valve Surgery in Octogenarians (MiSO), of high-volume specialist centers in the MitraClip (Abbott Vascular, Inc, Menlo Park, Calif) era.

## Patients and Methods

### Study Population and Definitions

MiSO is a multicenter, retrospective, observational study based on prospectively collected data obtained from institutional cardiac surgery datasets of 3 high-volume specialist UK centers (Bristol, Papworth, and Southampton). The study was conducted in accordance with the principles of the Declaration of Helsinki. Institutional board approval was obtained for the study, and patient consent was waived.

**Figure E1:**
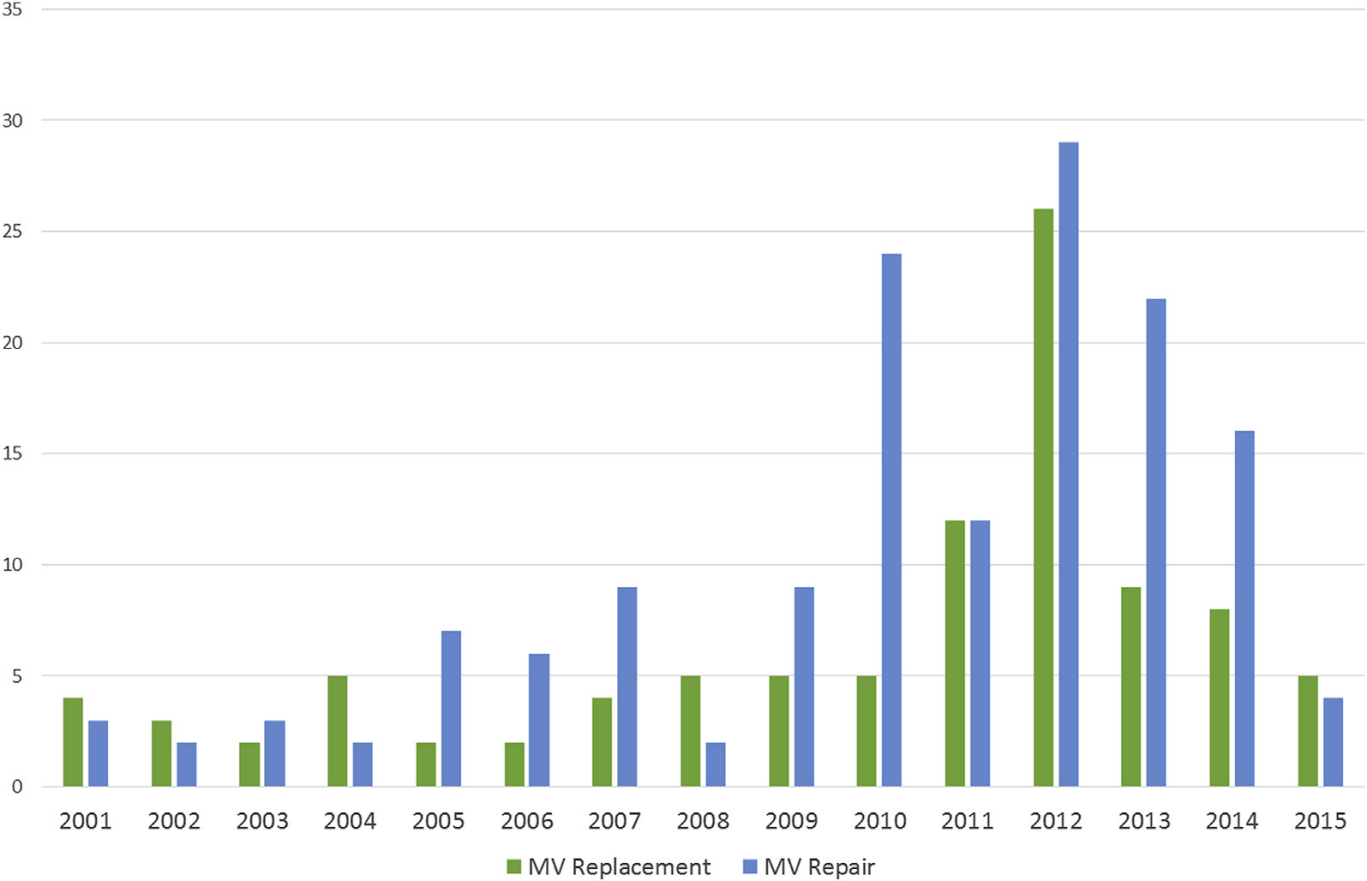
Distribution and type of mitral valve surgeries over the years. *MV*, Mitral valve.

Between January 2001 and October 2015, 252 octogenarians underwent mitral valve surgery. All the purely endocarditis, rheumatic, or ischemic cases were excluded to prevent selection bias. The final cohort consisted of 247 patients with degenerative MR (Figure E1 shows the distribution and type of surgery over the years). All patients were approached through a median sternotomy. Risk scoring has been calculated using the logistic European System for Cardiac Operative Risk Evaluation (euroSCORE). Degenerative disease was defined as single- or multi-segment prolapse due to chordal elongation or rupture. Emergency surgery was defined as surgery carried out within 24 hours of unscheduled admission, including patients presenting with acute decompensation of chronic MR. Patient demographics are summarized in Table 1. Table E1 shows the surgical techniques adopted.

**Table 1:** Preoperative characteristics

Characteristic	Surgical technique			
	Overall (247)	Repair (150)	Replacement (97)	*P* value
Age, y	82.8 ± 2.3	82.9 ± 2.3	82.6 ± 2.5	.12
Female gender	108 (43.7)	62 (41.3)	46 (47.4)	.41
BMI, kg/m^2^	24.3 ± 3.2	24.3 ± 3.6	24.4 ± 4.3	.89
LVEF				.87
Good (≥50%)	168 (68)	102 (68)	66 (68)	
Moderate impairment (≥30% and <50%)	64 (25.9)	38 (25.3)	26 (25.3)	
Severe impairment (<30%)	15 (6.1)	10 (6.7)	5 (6.7)	
Preoperative AF	113 (45.7)	79 (52.6)	34 (35.1)	<.01
Diabetes	13 (5.3)	8 (5.3)	5 (5.1)	1
Hypertension	133 (53.8)	80 (53.3)	53 (54.6)	.94
COPD	32 (13)	16 (10.7)	16 (16.5)	.98
Smoking history	113 (45.7)	71 (47.3)	42 (43.3)	.62
Previous MI	31 (12.6)	17 (11.2)	14 (14.4)	.76
Logistic euroSCORE	11.5 ± 13.4	10.2 ± 11.8	13.7 ± 15.2	.07
Previous CVA	19 (7.7)	11 (7.3)	8 (8.2)	.98
NYHA III/IV	180 (72.9)	106 (70.6)	74 (76.3)	.4
Previous cardiac surgery	17 (6.9)	6 (4)	11 (11.3)	.04
Emergency	6 (2.4)	2 (1.3)	4 (4.1)	.21
Urgency	34 (13.8)	23 (15.3)	11 (11.3)	.37

*BMI*, Body mass index; *LVEF*, left ventricular ejection fraction; *AF*, atrial fibrillation; *COPD*, chronic obstructive pulmonary disease; *MI*, myocardial infarction; *euroSCORE*, European System for Cardiac Operative Risk Evaluation; *CVA*, cerebrovascular accident; *NYHA*, New York Heart Association.

Left ventricular ejection fraction (LVEF) was classified into 3 groups: good (LVEF ≥50%), moderate (LVEF >30% and <50%), and poor (LVEF <30%). Patients were considered to have chronic obstructive pulmonary disease (COPD) if they had any of the following conditions: long-term use of bronchodilators or steroids for lung disease before admission; outpatient visits including a diagnosis of COPD on 2 occasions or a previous inpatient stay with a discharge diagnosis of COPD; and preoperative lung function test with evidence of obstructive pattern. Patients were considered to have diabetes if they had any of the following conditions: receipt of insulin or oral hypoglycemic medications before admission; outpatient visits including a diagnosis of diabetes mellitus on 2 occasions; or a previous inpatient stay with a discharge diagnosis of diabetes mellitus.

### End Points

The primary end points in the MiSO study were early mortality, defined as death due to all causes within 30 days from the day of surgery, and long-term survival. Secondary outcomes included new acute kidney injury (AKI) requiring dialysis, evidence of postoperative stroke (defined as clinical and radiologic evidence of a new postoperative cerebrovascular event), return to the operating room for cardiac causes, and sternal wound infection categorized in superficial (involving the skin, subcutaneous tissue, and pectoralis fascia only) and deep (involving sternal bone or mediastinal structures). Moreover, a composite outcome of death at 30 days or AKI with dialysis or postoperative cerebrovascular accident was considered for a multiple logistic regression analysis, designed to identify independent risk factors affecting early outcomes.

Data regarding long-term survival have been derived from our clinical dataset linked to the Office for National Statistics individually collected on October 31, 2015. The completeness for survival data was 100%.

### Statistical Analysis

Data are presented as mean ± 1 standard deviation for numeric continuous variables and as per total number and percentages for categoric variables. The numeric variables were tested for normality with the Shapiro–Wilk test. Comparison between numeric variables has been conducted with the Student *t* test for normally distributed variables or the Mann–Whitney *U* test for not normally distributed variables. Inference on categoric variable has been done using the chi-square or Fisher exact test. To identify the predictors of composite outcome, a multivariable logistic regression model was developed: The initial model included those variables that had a *P* value less than .25 with univariable analysis (age, previous cardiac surgery, intra-aortic balloon pump [IABP] use, emergency, logistic euroSCORE, concomitant coronary artery bypass grafting, reduced LVEF, and surgical technique): The final model was obtained by stepwise backward approach selecting for Akaike information criteria. Survival analysis was conducted using Kaplan–Meier methods and log-rank test. A Cox proportional hazard model was designed to identify independent factors affecting long-term survival. To estimate the predicting ability of logistic euroSCORE on 30-day mortality, we used receiver operating characteristics curve analysis. The area under the curve was estimated for observed euroSCORE and the predicted values by fitting regression model. Missing values were screened before analysis: Every variable with more than 5% of missing value was eliminated from the final dataset. The remaining variables (Table E2) were imputed using simple imputation methods. The statistical analysis was conducted with R statistical software (R version 3.1.2 [2014-10-31] —“Pumpkin Helmet”; R Foundation for Statistical Computing, Vienna, Austria).

## Results

A total of 150 patients (60.7%) underwent MVr, and 97 patients (39.3%) underwent MVR. The median follow-up was 2.9 years (minimum 0, maximum 14 years); mean follow-up was 41.4 ± 35.6 months. The distributions of baseline patient characteristics for the overall population are presented in Table 1. There were no significant differences in terms of age, body mass index (BMI), and other preoperative comorbidities between the 2 groups. The only significant differences were previous cardiac surgery (redo) (4% vs 11.3%, *P* = .04) and preoperative atrial fibrillation (52.6% vs 35.1%, *P* < .01) for the MVr and MVR groups, respectively. The New York Heart Association class distribution was similar in the 2 groups (Table 1). Patient operative risk, as estimated by the logistic euroSCORE, was lower in the MVr group (10.2 ± 11.8 vs 13.7 ± 15.2 in MVR), although this difference was not statistically significant (*P* = .07). No difference was found between groups for duration of cardiopulmonary bypass and aortic crossclamp times (Table 1). Associated procedures included 88 coronary artery bypass graftings (35.6%), 53 aortic valve procedures (21.5%), 47 tricuspid valve procedures (19%), and 19 (7.7%) atrial fibrillation ablation procedures. Operative characteristics and postoperative outcomes are summarized in Table 2. The 30-day mortality for the whole cohort was 13.8% (MVr 4.7% vs MVR 18.6%; *P* < .01). No differences were found in terms of postoperative cerebrovascular accident (MVr 2% vs MVR 3.1%; *P* = .9) and AKI requiring dialysis (6.7% vs 13.4%; *P* = .12). The overall incidence of sternal wound infection was not different between the 2 groups (12% vs 20.4%; *P* = .11). The deep sternal wound infection rate was not different (2% vs 3.1%, *P* = .67). The length of stay in hospital after the surgery was found to be significantly longer in the MVr group (14.1 ± 9.5 days vs 12.7 ± 14.7 days in MVR; *P* < .01).

**Table 2:** Operative characteristics and postoperative outcomes

Characteristic	Surgical technique			
	Overall (247)	Repair (150)	Replacement (97)	*P* value
CPB time (min)	133.5 ± 52.2	128.9 ± 47.5	140.5 ± 58.3	.21
Crossclamp time (min)	95.9 ± 38	91.1 ± 32.8	103.3 ± 44	.06
Concomitant procedures				
CABG	88 (35.6)	55 (36.6)	33 (34)	.77
AVR	53 (21.5)	27 (18)	26 (26.8)	.14
TV	47 (19)	30 (20)	17 (17.5)	.75
AF ablation	19 (7.7)	17 (11.3)	2 (2.1)	.02
30-d mortality	25 (13.8)	7 (4.7)	18 (18.6)	<.01
CVA	6 (2.4)	3 (2)	3 (3.1)	.9
AKI (dialysis)	23 (9.3)	10 (6.7)	13 (13.4)	.12
Sternal wound infection	37 (15.2)	18 (12)	19 (20.4)	.11
Superficial	31 (12.6)	15 (10)	16 (16.5)	.17
Deep	6 (2.4)	3 (2)	3 (3.1)	.67
Return to operating room	16 (6.5)	7 (4.7)	9 (9.2)	.24
Postoperative length of stay (d)	13.6 ± 11.9	14.1 ± 9.5	12.7 ± 14.7	<.01
Composite outcome (death at 30 d, AKI, CVA)	41 (16.6)	16 (10.6)	25 (25.7)	<.01

*CPB*, Cardiopulmonary bypass; *CABG*, coronary artery bypass grafting; *AVR*, aortic valve replacement; *TV*, tricuspid valve; *AF*, atrial fibrillation; *CVA*, cerebrovascular accident; *AKI*, acute kidney injury.

In the subgroup of patients who underwent a redo operation, the 30-day mortality rate was 16.7% in the repair group versus 18.2% in the replacement group (*P* = .9).

The final model logistic regression multivariable analysis (Table 3) for the short-term composite outcome showed that previous cardiac surgery (odds ratio [OR], 4.47; 95% confidence interval [CI], 1.37-17.46; *P* = .02), IABP use (OR, 4.77; 95% CI, 1.67-15.79; *P* < .01), and MVR (OR, 7.7; 95% CI, 4.04-14.9; *P* < .01) were independent negative predictors.

**Table 3:** Multivariable logistic regression model for predictors of short-term composite outcome (death at 30 days, acute kidney injury, and cerebrovascular accident)

Predictor	OR (95% CI)	*P* value
Previous cardiac surgery	4.47 (1.37-17.46)	.02
IABP use	4.77 (1.67-15.79)	<.01
Reduced LVEF	1.73 (0.88-3.43)	.11
MVR	7.7 (4.04-14.9)	<.01

*OR*, Odds ratio; *CI*, confidence interval; *IABP*, intra-aortic balloon pump; *LVEF*, left ventricular ejection fraction; *MVR*, mitral valve replacement.

The Cox proportional hazard model results (Table 4) showed MVR (hazard ratio [HR], 2.16; 95% CI, 1.42-3.3; *P* < .01) and IABP use (HR, 2.67; 95% CI, 1.52-4.7; *P* < .01) to be independent predictor factors affecting long-term survival. BMI was found to be a protective factor (HR, 0.95; 95% CI, 0.89-1.00; *P* = .06).

**Table 4:** Cox proportional hazard ratio multivariable model for predictor affecting long-term mortality∗

Predictor	HR (95% CI)	*P* value
BMI	0.94 (0.89-1.01)	.09
MVR	1.88 (1.22-2.89)	<.01
IABP use	2.54 (1.26-5.13)	<.01
Reduced LVEF	1.09 (0.42-2.82)	.84

*HR*, Hazard ratio; *CI*, confidence interval; *BMI*, body mass index; *MVR*, mitral valve replacement; *IABP*, intra-aortic balloon pump; *LVEF*, left ventricular ejection fraction.

∗ Gender has been included as stratum variable.

**Figure 1:**
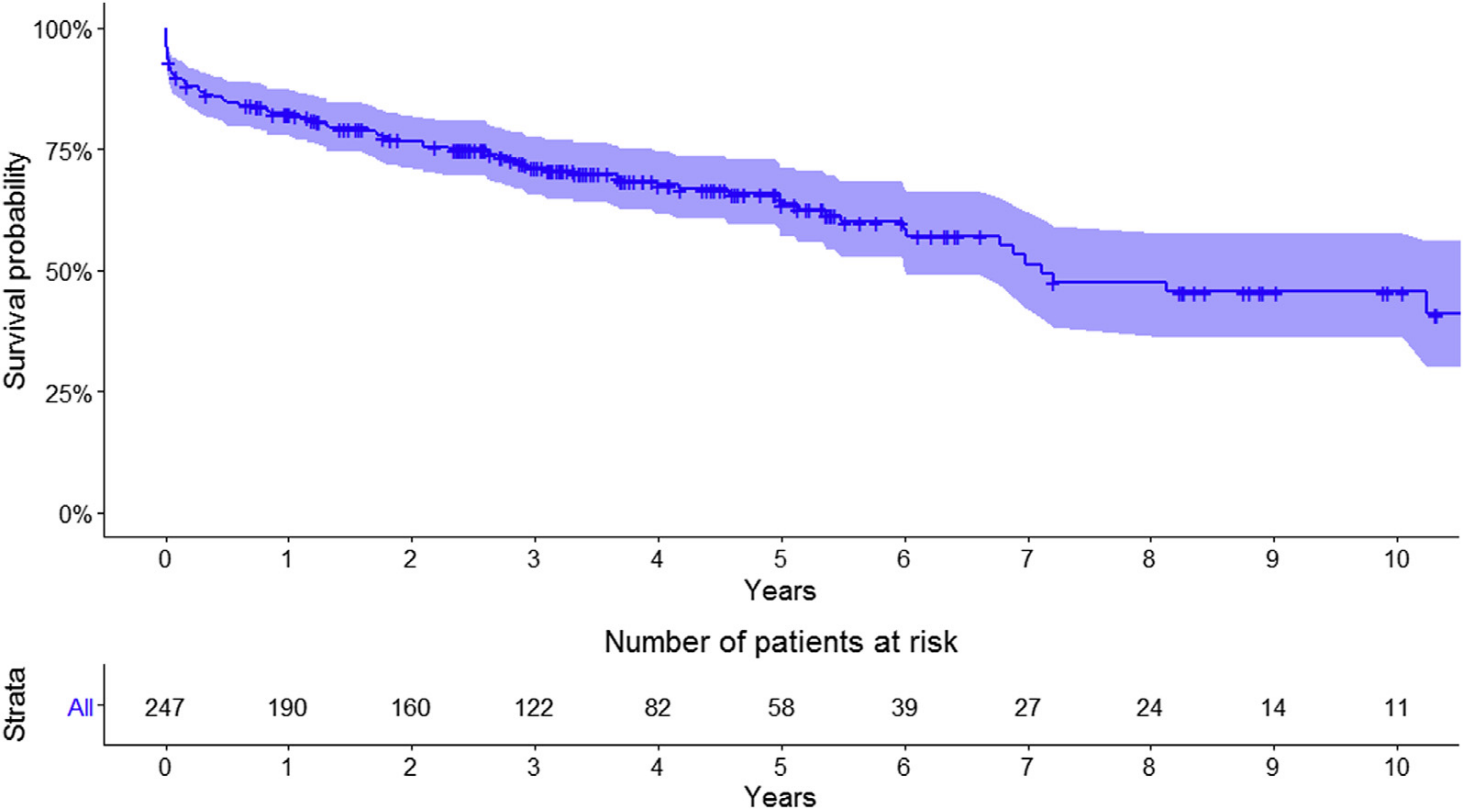
Kaplan–Meier survival curve for the overall surgical population.

**Figure 2:**
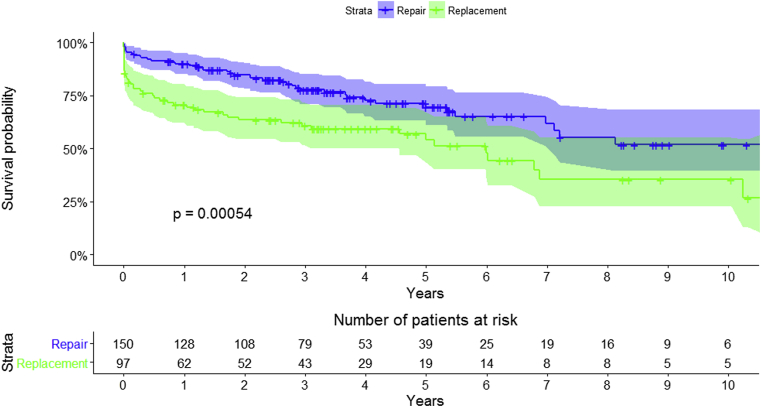
Kaplan–Meier survival curves between the 2 groups (raw data).

**Figure 3:**
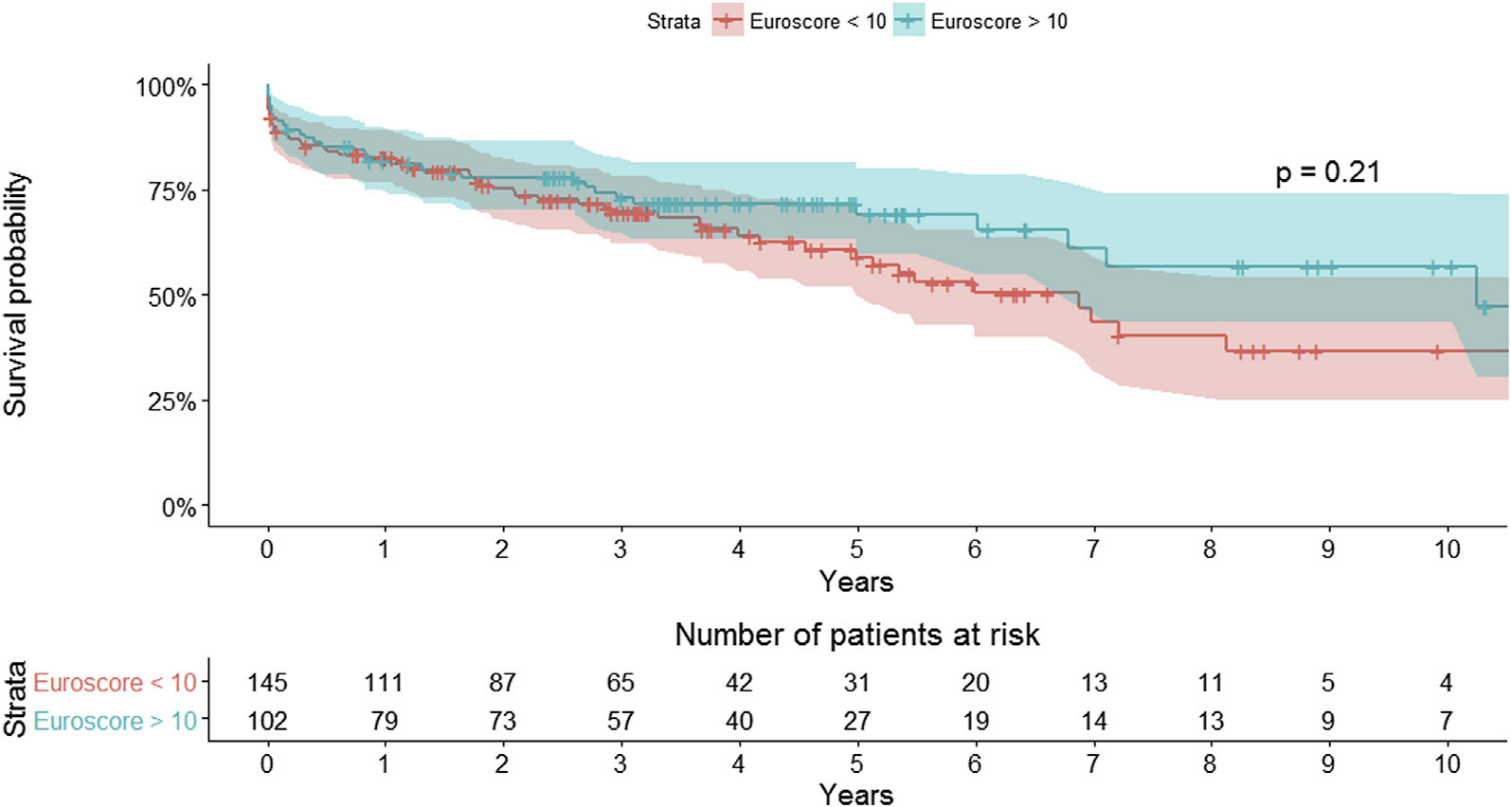
Impact of euroSCORE on survival: Kaplan–Meier survival curves. *euroSCORE*, European System for Cardiac Operative Risk Evaluation.

**Figure 4:**
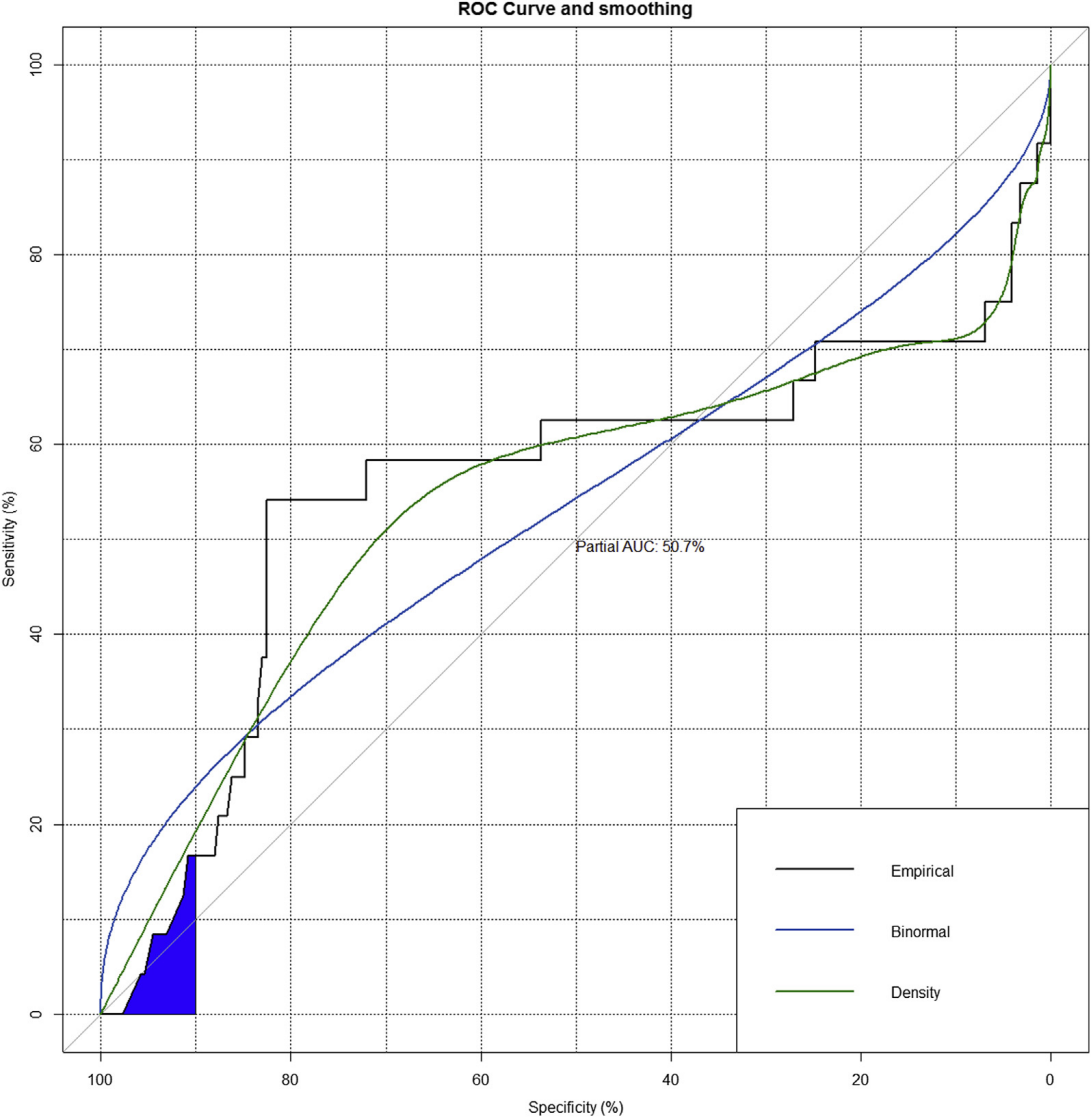
Predictive value of euroSCORE (receiver operating characteristics [*ROC*] curve). *AUC*, Area under the curve.

Overall survival for the entire cohort at 1, 5, and 10 years was 82.4% (95% CI, 77.8-87.3), 63.7% (95% CI, 57.0-71.2), and 45.5% (95% CI, 36.0-57.5): MVr versus MVR: 89.9% (95% CI, 85.2-94.9) versus 70.7% (95% CI, 62.1-80.5) at 1 year, 69.6% (95% CI, 61.2-79.1) versus 54.0% (95% CI, 43.5-67.1) at 5 years, and 51.8% (95% CI, 39.4-68.2) versus 35.5% (95% CI, 22.9-55.1) at 10 years, respectively (*P* < .01) (Figures 1 and 2). Logistic euroSCORE was unable to predict early and late mortality (area under the curve, 50.7%) (Figures 3 and 4).

## Discussion

In the MiSO study, we found that in octogenarians, MVr for degenerative disease is associated with good early outcomes and late survival. MVR is still associated with significant mortality. Furthermore, previous cardiac surgery and the use of perioperative IABP are associated with decreased long-term survival, and increased BMI seems to be protective. Logistic euroSCORE does not seem to effectively predict early outcome in this group of patients.

The last 2 decades have seen a growing proportion of octogenarian patients being referred for mitral valve surgery.^1^, ^2^, ^3^, ^4^ This increasing demand is related to prolonged life expectancy and reported better outcome of mitral surgery in this population.^3^, ^4^, ^5^, ^6^, ^7^, ^8^, ^9^, ^10^, ^11^ However, approximately half of patients affected by severe MR are not referred for surgery mainly because of their advanced age, comorbidities, and left ventricular dysfunction,^2^, ^4^ and in specialized centers there seems to be an increasing trend to treat elderly patients with percutaneous procedures.^27^, ^28^ This reflects the misperception that mitral valve surgery is associated with prohibitively high early mortality in this age group. The decision for surgery is complex; several elements, such as the lack of correspondence between physiologic and chronologic age, the quality of life, and the risk–benefit ratio, should be taken into account before confirming a surgical indication. This article, analyzing the early and long-term outcomes of 247 octogenarians undergoing conventional mitral valve surgery, is aimed at assisting in making an informed decision for the patients and the clinicians regarding surgery or catheter-based mitral intervention in octogenarian patients with degenerative mitral valve disease.

The results of our study suggest that being octogenarians does not necessarily implicate higher surgical risk. With the help of receiver operating characteristics analysis, we have confirmed the suspicion that the logistic euroSCORE does not provide a valid means of determining the predicted mortality in this unique subset of patients, and there is a need to generate a mitral score on the basis of large multicenter mitral valve surgery registries, especially for very high-risk patients.

In our study, 30-day mortality for the whole cohort was 13.8% (MVr 4.7% vs MVR 18.6%; *P* < .01). This result is similar to reports by other series; the operative mortality in a cohort of 2700 octogenarian patients undergoing MVR between 1997 and 2000 from the Society of Thoracic Surgeons National Cardiac Database was 16.9%.^13^ In smaller single-center studies, operative mortality has ranged from 15% to 20%,^13^, ^14^, ^17^, ^19^, ^20^, ^22^ which is similar to what we observed in our overall cohort. However, lower operative mortality has been reported in elderly patients with nonischemic MR undergoing isolated mitral surgery similar to that reported in this article. Moreover, with an overall survival of 63.7% at 5 years and 45.5 at 10 years, we showed that this surgery has important long-term benefits even in octogenarians, with a substantial superiority of MVr compared with MVR. It has been shown that MVr is preferred over MVR to treat MR due to degenerative mitral valve disease with improved short- and long-term survivals.^5^, ^6^, ^7^, ^8^, ^9^, ^10^, ^11^ Initially, MVr was considered to be an unnecessary procedure in elderly subjects; moreover, there were concerns that fragile tissues and valvular calcification were more likely to preclude satisfactory valve repair in older patients.^14^ However, with increasing skill and understanding of mitral disease, the repair rate, especially in expert hands in high-volume centers, has continued to grow in the last few years.^12^, ^13^, ^14^, ^15^, ^16^, ^17^, ^18^, ^19^, ^20^, ^21^, ^22^, ^23^, ^24^, ^25^, ^26^ Our study confirms this trend, with an increasing ratio of MVr to MVR over time in octogenarians without significant cardiopulmonary bypass time difference between the 2 techniques. In the octogenarian population, a survival of approximately 70% at 5 years and approximately 52% at 10 years after MVr for degenerative MR in our high-volume multicenter experience strengthens the case to recommend MVr as the gold standard for degenerative MR in octogenarians.

An increasingly emerging alternative in treating mitral valve disease is represented by the minimally invasive approach through right minithoracotomy. Faster recovery and reduced pain make this approach a valid and reliable substitute to classic median sternotomy.^33^, ^34^, ^35^, ^36^, ^37^, ^38^ There is a large body of evidence in the literature showing that minimally invasive mitral valve surgery is not inferior to the conventional surgical approach using a full sternotomy, especially with regard to operative results and long-term survival.^34^, ^35^ This seems to be valid even in octogenarians, with some authors reporting a trend toward a lower operative mortality, rate of transfusion, and an overall faster recovery with shorter postoperative hospital stays for the minimally invasive approach.^36^, ^37^, ^38^

Transcatheter mitral technologies have developed as a viable option to treat high-risk and inoperable patients with severe MR, having shown promising early outcomes, especially in terms of procedural safety.^27^, ^28^, ^29^, ^30^, ^31^, ^32^ Buzzatti and colleagues^30^ recently published their results comparing the outcomes of patients aged 80 years or more, affected by isolated degenerative MR, who underwent isolated transcatheter (n = 25) or surgical (n = 35, 29 repairs and 6 replacements) mitral intervention. Follow-up overall mortality was higher in the MitraClip group (MitraClip System, Abbott Vascular, Inc) than in the surgery group, although after the first 30 days, only 1 of 5 deaths observed in the MitraClip group was cardiac related. Regardless of survival, as confirmed by Lim and colleagues^31^ in a population of patients with prohibitive-risk degenerative MV, the MitraClip can make octogenarians with severe degenerative MR feel better, reducing their symptoms and improving their quality of life. A recent meta-analysis by Takagi and colleagues^32^ comparing the MitraClip with surgical repair for MR in all age groups showed similar early and late mortality survival between the 2 procedures, despite higher-risk profiles in the percutaneous intervention group.

An interesting finding of this study is represented by the protective effect of BMI. There is increasing evidence regarding the protective role of obesity in cardiac surgery.^39^, ^40^ In an apparent paradox, morbidity and mortality are lower in obese patients undergoing cardiac surgery, although the nature of this association is unclear. Even in our subgroup of octogenarians undergoing mitral valve surgery, this protective effect was confirmed.

### Study Limitations

This study is retrospective and multicenter, and thus has the associated limitations of potential heterogeneity. It includes a small cohort of patients, and because of the logistics, it was not possible to achieve long-term clinical echocardiographic follow-up for all the 3 centers. Moreover it lacks data on the indication for repair or replacement at the time of surgery and therefore is unable to correctly delineate factors affecting surgical selection, and also does not provide information on the long-term cause of death. The absence of a nonsurgical group (ie, medical treatment or transcatheter treatment) is another important limitation.

## Conclusions

In the MiSO study involving high-volume mitral specialist centers, we have demonstrated that in octogenarians, MVr for degenerative disease is associated with very good survival and should be considered the gold standard. Predictors of poor long-term survival include previous cardiac surgery and the need for perioperative IABP. Larger international multicenter registries are required to create a powerful scoring system in guiding decision-making in such a high-risk subgroup because the logistic euroSCORE does not accurately reflect the exact risk profile of these patients.

### Conflict of Interest Statement

Authors have nothing to disclose with regard to commercial support.

## Figures and Tables

**Table E1:** Surgical characteristics

Characteristic	Surgical technique	
	Repair (150)	Replacement (97)
Repair type∗		
Leaflet resection	43 (37.1)	NA
Annuloplasty ring	116 (100)	NA
Neochordae	43 (37.1)	NA
Replacement		
Mechanical	NA	10 (10.3)
Biological	NA	87 (89.7)
Prostheses size		
23 mm	NA	2
25 mm	NA	16
27 mm	NA	20
29 mm	NA	26
31 mm	NA	18
33 mm	NA	15
Procedures per center		
Center A	58	23
Center B	40	35
Center C	52	39

*NA*, Not available.

∗ Data available for 116 patients (77.3% of the total).

**Table E2:** Missing values distribution

Characteristic	Count of missing values	% of missing values
Age, y	0	0
Female gender	0	0
BMI, kg/m^2^	0	0
LVEF	3	1.2
Preoperative rhythm	0	0
Diabetes	1	0.4
Hypertension	1	0.4
COPD	1	0.4
Smoking history	1	0.4
Previous MI	2	0.8
Logistic euroSCORE	5	2
Previous CVA	2	0.8
NYHA III/IV	0	0
Previous cardiac surgery	1	0.4
Operative urgency	0	0
CPB time	2	0.8
Crossclamp time	2	0.8
Concomitant procedures	1	0.8
30-d mortality	0	0
CVA	4	1.6
AKI (dialysis)	2	0.8
Sternal wound infection	0	0
Return to operating room	0	0
Postoperative length of stay (d)	0	0

*BMI*, Body mass index; *LVEF*, left ventricular ejection fraction; *COPD*, chronic obstructive pulmonary disease; *MI*, myocardial infarction; *euroSCORE*, European System for Cardiac Operative Risk Evaluation; *CVA*, cerebrovascular accident; *NYHA*, New York Heart Association; *CPB*, cardiopulmonary bypass; *AKI*, acute kidney injury.
